# Highly Luminous Ba_2_SiO_4−δ_N_2/3δ_:Eu^2+^ Phosphor for NUV-LEDs: Origin of PL-Enhancement by N^3−^-Substitution

**DOI:** 10.3390/ma13081859

**Published:** 2020-04-15

**Authors:** Donghyeon Kim, Tae Hun Kim, Tae Eun Hong, Jong-Seong Bae, Chang Hae Kim, Jaegyeom Kim, Seung-Joo Kim, Ki-Wan Jeon, Jung-Chul Park

**Affiliations:** 1 Department of Energy Systems Research and Department of Chemistry, Ajou University, Suwon 16499, Korea; minam8358@gmail.com (D.K.); jaegyeom86@gmail.com (J.K.); sjookim@ajou.ac.kr (S.-J.K.); 2 Department of Engineering in Energy and Applied Chemistry, Silla University, Busan 46958, Korea; younwoo444@naver.com (T.H.K.); kiwan@silla.ac.kr (K.-W.J.); 3 Busan Center, Korea Basic Science Institute, Busan 46742, Korea; tehong@kbsi.re.kr (T.E.H.); jsbae@kbsi.re.kr (J.-S.B.); 4 Advanced Materials Division, Korea Research Institute of Chemical Technology (KRICT), 141, Gajeong-ro, Yuseong-gu, Daejeon 34114, Korea; changhae@krict.re.kr

**Keywords:** Ba_2_SiO_4_:Eu^2+^, N^3−^ substitution, SIMS, IR, XPS, PL

## Abstract

Ba_2_SiO_4−δ_N_2/3δ_:Eu^2+^ (BSON:Eu^2+^) materials with different N^3−^ contents were successfully prepared and characterized. Rietveld refinements showed that N^3−^ ions were partially substituted for the O^2−^ ions in the SiO_4_-tetrahedra because the bond lengths of Si‒(O,N) (average value = 1.689 Å) were slightly elongated compared with those of Si‒O (average value = 1.659 Å), which resulted in the minute compression of the Ba(2)‒O bond lengths from 2.832 to 2.810 Å. The average N^3−^ contents of BSON:Eu^2+^ phosphors were determined from 100 nm to 2000 nm depth of grain using a secondary ion mass spectrometry (SIMS): 0.064 (synthesized using 100% α-Si_3_N_4_), 0.035 (using 50% α-Si_3_N_4_ and 50% SiO_2_), and 0.000 (using 100% SiO_2_). Infrared (IR) and X-ray photoelectron spectroscopy (XPS) measurements corroborated the Rietveld refinements: the new IR mode at 850 cm^−1^ (Si‒N stretching vibration) and the binding energy at 98.6 eV (Si-2p) due to the N^3-^ substitution. Furthermore, in UV-region, the absorbance of N^3−^-substituted BSON:Eu^2+^ (synthesized using 100% α-Si_3_N_4_) phosphor was about two times higher than that of BSO:Eu^2+^ (using 100% SiO_2_). Owing to the N^3−^ substitution, surprisingly, the photoluminescence (PL) and LED-PL intensity of BSON:Eu^2+^ (synthesized using 100% α-Si_3_N_4_) was about 5.0 times as high as that of BSO:Eu^2+^ (using 100% SiO_2_). The compressive strain estimated by the Williamson−Hall (W−H) method, was slightly increased with the higher N^3−^ content in the host-lattice of Ba_2_SiO_4,_ which warranted that the N^3-^ ion plays an important role in the highly enhanced PL intensity of BSON:Eu^2+^ phosphor. These phosphor materials could be a bridgehead for developing new phosphors and application in white NUV-LEDs field.

## 1. Introduction

Recently, light-emitting diodes (LEDs) have been rapidly developed as solid state lighting sources as well as components in flat-panel displays. Commercial white LEDs were initially developed using the combination of blue-emitting InGaN chips and a YAG:Ce^3+^ phosphor, resulting in the white light-emission [1]. Although this LED exhibits high efficiency, the color rendering index is poor. As the phosphor materials play an important role in generating human-friendly white light, it is essential to develop the improved color rendering index phosphor materials. So, many researchers have given much attention to the development of new phosphors for white LEDs in the last decade. With respect to aforementioned, rare earth activated metal silicon-oxynitride phosphors have been considered as a breakthrough to improve the low color rendering index for white LED applications [2,3,4,5,6,7,8]. Divalent europium ions (Eu^2+^) stabilized in the metal silicon-oxynitride phosphors, have been used as a very useful activator that exhibits the broad emission bands between the ultraviolet (UV) and red spectral range corresponding to 4f^6^5d^1^–4f^7^ transition [7,8,9]. The partially substituted N^3−^ ions for O^2−^ ions in the alkaline earth silicate lattice might change the electronic structure of Eu^2+^ ions, which could be induced by different electronegativity, crystal field splitting, and nephelauxetic effect [10], resulting in the change of the luminescence properties of the metal silicon-oxynitride phosphors. Additionally, a point view of the inorganic chemistry, the contents of the substituted N^3−^ ions for O^2−^ ions are of importance for understanding the luminescence properties of the metal silicon-oxynitride phosphors. Attempts to partial replacement of O^2−^ ions with N^3−^ ions were carried out [2,3,4,5,6,7,8,9], however, there has been no report on the systematic research for origin of the enhanced PL property as well as an effect of the N^3−^ ion contents in metal silicon-oxynitride phosphors, such as BaSi_2_O_2−δ_N_2+2/3δ_ [2], Ba_4−z_M_z_Si_8_O_20−3x_N_2x_ [6], and Ba_2_Si_5−x_Al_x_N_8−x_O_x_ [8].

The present work reports on the syntheses and characterization of green-emitting Ba_2_SiO_4-δ_N_2/3δ_:Eu^2+^ (BSON:Eu^2+^) phosphor in comparison to Ba_2_SiO_4_:Eu^2+^ (BSO:Eu^2+^). The contents of N^3−^ ions in the green-emitting BSON:Eu^2+^ phosphor are quantitatively determined using secondary ion mass spectrometry (SIMS). In particular, the origin of the remarkably enhanced luminescence for BSON:Eu^2+^ phosphor is verified using infrared (IR), X-ray photoelectron spectroscopy (XPS), UV/Visible absorbance, photoluminescence (PL) and LED-PL.

## 2. Experimental

BSON:Eu^2+^ phosphors were synthesized with a mixture of BaCO_3_, α-Si_3_N_4_, and Eu_2_O_3_ under NH_3_ atmosphere at 1150 °C for 6 h. Typically, a stoichiometric amount of BaCO_3_, α-Si_3_N_4_, and Eu_2_O_3_ powder was ground in an agate mortar and pestle to obtain a fine and well mixed powder, which was then transferred to an alumina crucible. The alumina crucible containing the mixed precursors was then placed in a tube furnace and gradually heated at 5 °C/min to 1150 °C, where it was kept for 6 h under a flow of NH_3_ gas, and cooled to room temperature, producing BSON:Eu^2+^ phosphors. BSO:Eu^2+^ phosphors were synthesized with a mixture of BaCO_3_, SiO_2_, and Eu_2_O_3_ under 4%-H_2_/Ar gas at 1250 °C for 6 h. The contents of N^3−^ ion were controlled with different ratios between α-Si_3_N_4_ and SiO_2_, i.e., using only α-Si_3_N_4_, a mixture of α-Si_3_N_4_ and SiO_2_ (1:1 Si atomic %), and only SiO_2_. X-ray diffraction (XRD) patterns were obtained on a Rigaku D/MAX-2500 diffractometer (Rigaku corporation, Tokyo, Japan) using Cu-K_α_ radiation (λ = 1.5418 Å) with graphite-monochromator. Data were collected in the 2θ range between 5° and 110° for 4 s at 0.02° steps in room temperature. Refinements of the structural parameters were carried out using Fullprof software (Version 6.10). For the Rietveld refinements, the data of the structural parameters of single crystal-Ba_2_SiO_4_ [11] were used. The shape of the diffraction peaks was modeled with the pseudo-Voigt function. In the refinements, a manual background correction was used. SIMS (CAMECA IMS-6f, Gennevilliers, France) was used to analyze the elemental composition and contents of N ion in phosphors. The SIMS standard sample was synthesized using SiO_2_ film implanted by N^+^ at 100 keV at a dose of 5 × 10^14^ ions/cm^2^. In order to determine the relative sensitivity factor (RSF) of each element, the secondary ion intensity of each element was corrected by the SIMS results of the standard sample. For high precision measurements, focused Cs^+^ primary ion beam was used with an electron neutralizer for charge compensation (net impact energy = 15 keV, beam current = 20 nA). Field emission scanning electron-microscopy (FE-SEM) was performed using a Hitachi (Tokyo, Japan) S-4200 electron microscope operating at 15 kV. Fourier-transform infrared spectroscopy (FT-IR) was carried out using IRTracer-100 (Shimadzu corporation, Kyoto, Japan) in the range 400–2000 cm^−1^ using a KBr medium (the resolution range of ± 0.5 cm^−1^). The doped N-ions were also identified by X-ray photoelectron spectroscopy (XPS) (model: ESCALAB 250) with a Al K_α_ monochromator (*hν* = 1486.6 eV) at Busan Center of Korea Basic Science Institute (KBSI). The measured binding energies (BEs) were corrected with the adventitious carbon (C 1s) core level peak at 284.6 eV as an internal reference material. The PL quantum yields of phosphor materials before and after N^3−^ substitution were measured using a quantum yield measurement system with an integrating sphere (Hamamatsu Photonics, Hamamatsu, Japan, model: C9920-02) at ambient temperature.

## 3. Results and Discussion

### 3.1. Rietveld Refinements

**Figure 1:**
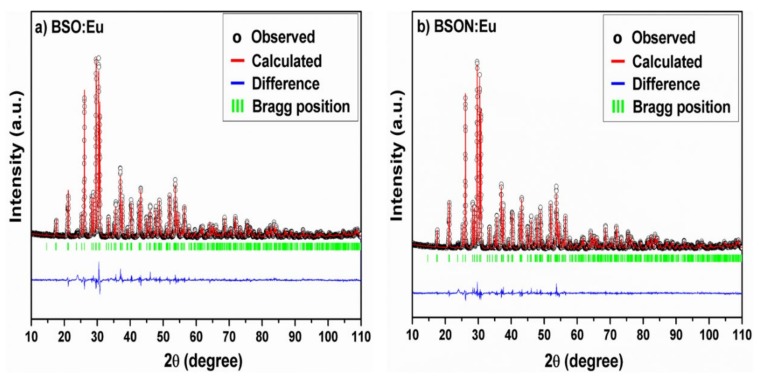
Rietveld refinement profiles of the X-ray diffraction (XRD) patterns for BSO:Eu (**a**) and BSON:Eu (**b**).

Figure 1 shows the powder XRD patterns and refinements of BSO:Eu and BSON:Eu.

**Figure 2:**
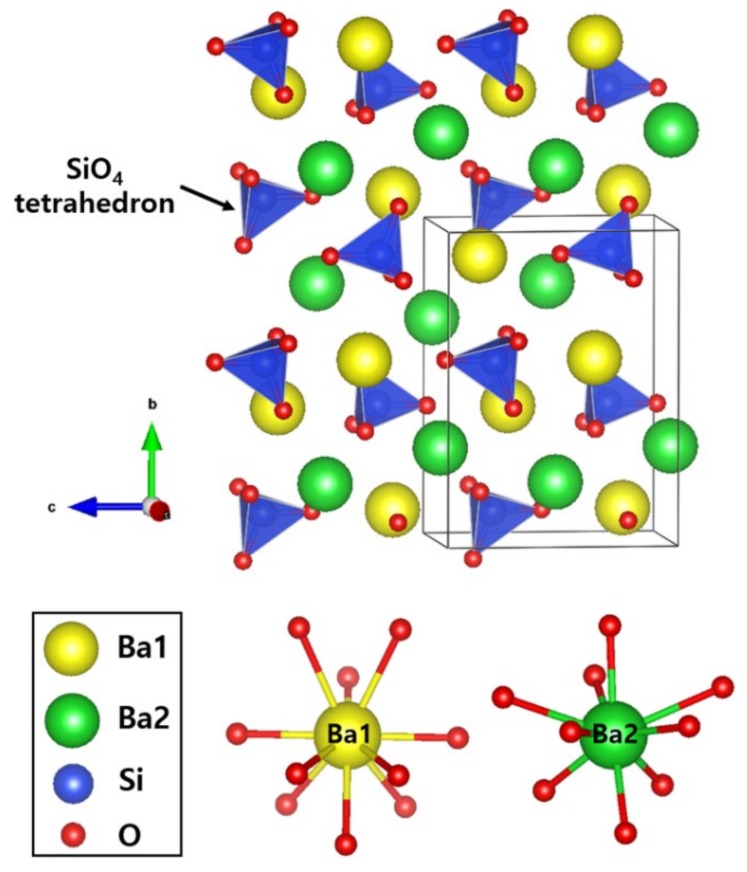
Unit-cell structure of Ba_2_SiO_4_ host-lattice.

The two samples have a primitive orthorhombic cell (space group = *Pmcn*) [11]. While performing Rietveld refinements, it was assumed that an equal amount of Eu atoms occupied two crystallographically distinct Ba sites in the lattice of Ba_2_SiO_4_ as depicted in Figure 2. 

The structural parameters extracted from the Rietveld fitting using the XRD data are given in Table 1. 

According to the XRD analysis, the XRD patterns of two samples are nearly identical. The difference of the unit cell size after N-doping was observed. Although the ionic radius of N^3−^ ion (1.46 Å) is larger than that of O^2−^ ion (1.38 Å) in coordination number (CN) = 4, the lattice parameters of BSON:Eu are slightly shortened by introducing the nitrogen atom, and thus, the unit cell volume is slightly decreased from 444.65 to 443.96 Å^3^ by N ion doping. A possible reason for this volume reduction can be deduced from the partial substitution of Si$O_{4}^{4-}$ groups by N^3−^ ions. This complex substitution would increase the structural disorder. The comparatively larger R factors observed in Rietveld refinement for N-doped BSON:Eu phosphor can support this hypothesis. The bond lengths and bond angles are listed in Table 2. 

As N^3−^ ions are introduced into the O^2−^ ion sites of SiO_4_ tetrahedrons, the bond lengths of Si‒O(1) and Si‒O(3) are slightly elongated from 1.681 to 1.752 Å and from 1.636 to 1.681 Å, respectively. In contrast, the bond length of Si‒O(2) is slightly shortened from 1.682 Å to 1.642 Å after N^3-^ ion doping. In terms of competition of bonding character, the bond lengths of Ba‒O are under minute compression after N^3-^ ion doping, from 2.975 to 2.970 Å for Ba(1)–O and from 2.832 to 2.810 Å for Ba(2)‒O. This result obtained by Rietveld refinements implies that N ions are very slightly doped into the lattice sites of Ba_2_SiO_4_.

### 3.2. Secondary Ion Mass Spectrometry: Average N^3−^ Content of BSON:Eu^2+^

**Figure 3:**
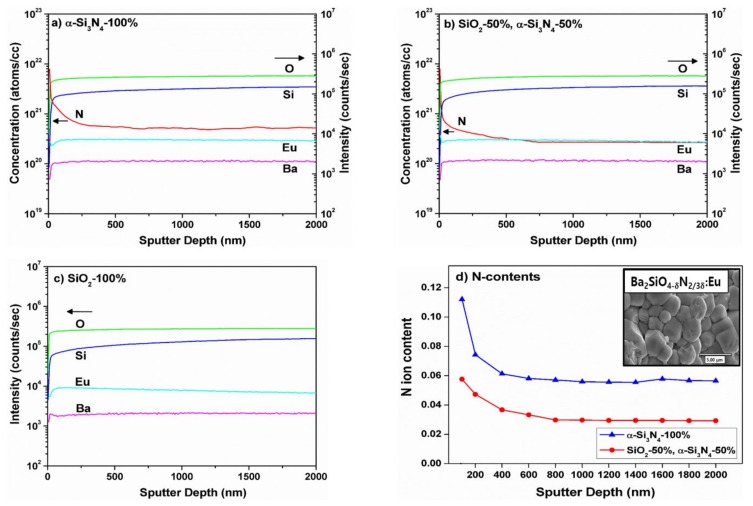
Atomic concentration and intensity as a function of sputtered depth of BSON:Eu^2+^ phosphor synthesized using α-Si_3_N_4_-100% (**a**), SiO_2_–50% and α-Si_3_N_4_–50% (**b**), SiO_2_–100% (**c**), and determined N ion contents (**d**) by secondary ion mass spectrometry (SIMS).

It is well-known that SIMS offers the best detection limit among all of the surface-analytical techniques, which allows us to analyze the present elements despite at extremely low concentrations. The elemental composition determined by SIMS can be quantified using a standard implanted by ion with a known dose [12,13,14]. The atomic concentration and intensity as a function of the sputtered depth of BSON:Eu^2+^ and BSO:Eu^2+^ phosphor are presented in Figure 3. 

Any distinct difference was not observed in the secondary ion intensities (for Ba, Eu, Si, and O atom) from the surface to the core of the grains for three phosphors, implying that each particle is elementally homogeneous throughout the entire particle, as desired. The N atom concentrations were measured from surface to the core of the particle. For the N^3−^-substituted BSON:Eu phosphor prepared with α-Si_3_N_4_ solely used (Figure 3a), the N atom concentration suddenly reduced near the surface (up to 200 nm) and constantly maintained around 600 nm in depth. In the preparing process, α-Si_3_N_4_ (the starting chemical) and NH_3_ gas (reaction gas) were used to substitute N^3−^ ions for O^2−^ ions. Presumedly, the N atoms from NH_3_ gas could diffuse from the surface to the core of the particles and those from α-Si_3_N_4_ correspond to the atomic concentration of N atom in the vicinity of 600 nm in depth with a constant value. To verify our hypothesis, we synthesized a phosphor with BaO, Eu_2_O_3_, and α-Si_3_N_4_ at 1150 °C under NH_3_ atmosphere as a control experiment, resulting in atomic rearrangement between O and N atoms, so the concentration of N atoms seems to have an effect on the chemical bonding character, i.e., the covalency (or ionicity) of Si‒N and Si‒O bond, which is closely related to the change of the Eu^2+^ electronic structure. For the N^3−^ -doped BSON:Eu phosphor prepared using a mixture of SiO_2_ and α-Si_3_N_4_ (1:1 Si atomic %) (see Figure 3b), the concentration of N atom was slightly lower than the one prepared using only α-Si_3_N_4_, implying that the N atom concentrations of phosphors are increased in proportion to the amount of α-Si_3_N_4_ used in our preparation step. Figure 3d shows the determined N ion concentration using the unit cell volume of BSON:Eu^2+^ phosphor. The contents of N ions are gradually decreased from the surface to 600 nm in depth of BSON:Eu^2+^ phosphor, then remained to be nearly constant values. As shown in the inset of Figure 3d, it is clearly seen that the average grain size is about 3 μm with pseudo-spherical grains. The average N ion contents of BSON:Eu^2+^ phosphors synthesized using only α-Si_3_N_4_, a mixture of α-Si_3_N_4_ and SiO_2_ (1:1 Si atomic %) were estimated as 0.064, 0.035 from 100 nm to 2000 nm in depth of the particle, respectively. 

### 3.3. Infrared Spectroscopy: The Evidence of N^3−^ Substitution

**Figure 4:**
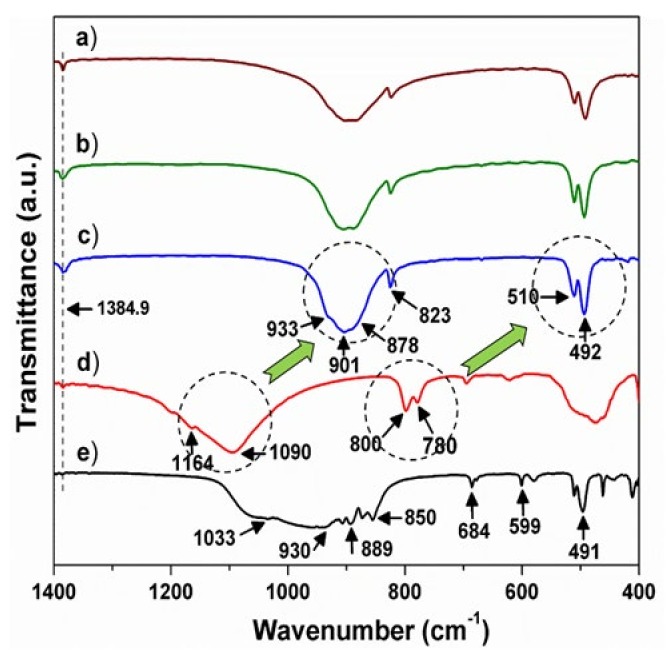
Infrared (IR) spectra of BSON:Eu^2+^ phosphor synthesized using α-Si_3_N_4_–100% (**a**), SiO_2_–50% and α-Si_3_N_4_–50% (**b**), SiO_2_–100% (**c**), SiO_2_ (after firing at 1250 ℃ under 4%–H_2_/Ar) (**d**), and α-Si_3_N_4_ (**e**).

Figure 4 presents the IR spectra of BSO:Eu and BSON:Eu, including SiO_2_ (after firing at 1250 °C under 4%-H_2_/Ar), and α-Si_3_N_4_. 

**Figure 5:**
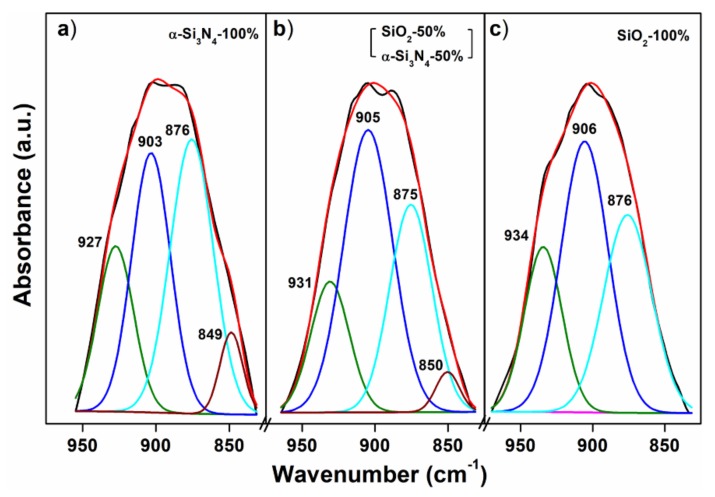
Gaussian fitting of vibration bands of BSO:Eu^2+^ and BSON:Eu^2+^ phosphor between 830 and 970 cm^−1^: α-Si_3_N_4_–100% (**a**), SiO_2_–50% and α-Si_3_N_4_–50% (**b**), SiO_2_–100% (**c**).

The vibration bands around 1400 cm^−1^ in all compounds were observed at exactly the same region (at 1384.9 cm^−1^), which corresponds to the C–O antisymmetric stretching of the ${\mathrm{CO}}_{3}^{2-}$ adsorbed on the surface of the particles [15,16,17]. Thus, it can be assured that the vibration peak at 1384.9 cm^−1^ can be considered as an internal standard to calibrate the wavenumber of the IR modes in the compounds. In the Ba_2_SiO_4_ structure, Si$O_{4}^{4-}$ complex anions are isolated from each other, but are linked by Ba^2+^ ions and the Si‒O bond length (average value = 1.665 Å) is shorter relatively to that of Ba‒O (average value = 2.903 Å). So, the internal modes of [SiO_4_] are mainly manifested between 1200 and 400 cm^−1^. By comparing SiO_2_ and α-Si_3_N_4_, it can be assured that for α-Si_3_N_4_, the vibration bands at 930 cm^−1^ corresponding to the Si‒N‒Si stretching mode [18,19,20] shift to a lower frequency region compared with those around 1090 cm^-1^ related with the Si‒O stretching mode [21,22,23]. This chemical shift between SiO_2_ and α-Si_3_N_4_ can be clearly elucidated by the different bond distance (1.62 Å for SiO_2_, 1.73 Å for α-Si_3_N_4_) [24,25] and the distinct bond energy (454 kJ/mol for SiO_2_, 426 kJ/mol for α-Si_3_N_4_) [26]. In the barium orthosilicate, Ba^2+^ ions occupy interstices in which they are linked by O^2-^ ions of the isolated Si$O_{4}^{4-}$ ions. As a result of the chemical bonding (Ba^2+^—[O‒Si‒O]^4-^—Ba^2+^) [27], the Si‒O bond strength weakens, giving rise to the blue-shift of frequency of the [SiO_4_] vibration modes in BSON:Eu and BSO:Eu, which is relative to those of SiO_2_ as shown in Figure 4a–c. As presented in Figure 4c, the vibration bands at 901 cm^−1^ are assigned to the Si‒O stretching, and those around 500 cm^−1^ are assigned to the O‒Si‒O bending, which is consistent with the previously reported results [28,29]. Particularly, it is clearly observed that the vibration modes at 800 cm^−1^ in SiO_2_ (Figure 4d) are shifted to the region around 500 cm^−1^ in BSO:Eu (Figure 4c) considering the shape and Δῡ of modes (Δῡ = 20 cm^−1^ for SiO_2_, Δῡ =18 cm^−1^ for BSO:Eu). To identify Si‒N bonding property in BSON:Eu phosphors, all of the IR spectra between 970 cm^−1^ and 830 cm^−1^ (in three phosphors with different N^3−^ contents) are deconvoluted by Gaussian fitting and are shown in Figure 5. 

After curve fitting, the new band around 850 cm^−1^ is dramatically noticeable in two phosphors synthesized using only α-Si_3_N_4_ and a mixture of SiO_2_ and α-Si_3_N_4_, whereas no band around 850 cm^−1^ is observed in the phosphor using only SiO_2_ as shown in Figure 5. Moreover, after curve fitting, three main peaks (around 930 cm^−1^, 900 cm^−1^, 870 cm^−1^) can be assigned to the Si‒O stretching modes [28,29]. Thus, the new peak around 850 cm^−1^ can be assigned to the Si‒N stretching mode and the bond length between SiO_2_ and α-Si_3_N_4_ (1.62 Å for SiO_2_, 1.73 Å for α-Si_3_N_4_) is obviously differentiated [24,25]. Notably, to the best of our knowledge, the Si‒N vibration mode is for the first time confirmed in the barium silicon-oxynitride phosphors. 

### 3.4. X-Ray Photoelectron Spectroscopy: The Evidence of N^3−^ Substitution 

**Figure 6:**
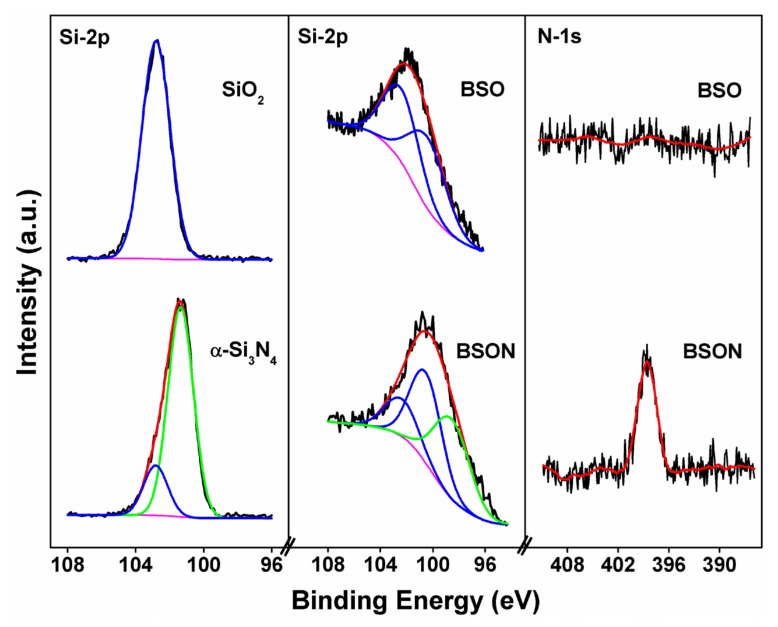
Si-2p and N-1s X-ray photoelectron spectroscopy (XPS) spectra of SiO_2_, α-Si_3_N_4_, BSO, and BSON compounds.

The formation of the chemical bond, Si‒N in the Ba_2_SiO_4_ crystal lattice, could be identified by the XPS technique. Figure 6 shows the Si-2p binding energies of SiO_2_, α-Si_3_N_4_, BSO, and BSON compounds. All spectra were adjusted with a Shirley background correction. 

As presented in Figure 6, the Si-2p binding energy (102.8 eV) in SiO_2_ as a reference material was observed at 102.8 eV with a single Gaussian component (FWHM = 1.9 eV), indicating the identical chemical environment of silicon atoms, whereas the Si-2p binding energy in α-Si_3_N_4_ as a reference material were fitted by two Gaussian components; 101.4 eV and 102.8 eV corresponding to the Si-2p of α-Si_3_N_4_ and SiO_2_ (α-quartz), respectively.

It should be mentioned that the latter binding energy (102.8 eV) of Si-2p may be due to the minute surface oxidation induced by the X-ray radiation or air atmosphere as its intensity is very low. Interestingly, it was reported that the Si-2p binding energy in SiC, Si_3_N_4_, and SiO_2_ was about 100.4 eV [30,31], 101.8 eV [32,33], 102.9 eV [33,34], respectively. The Si-2p binding energy in three compounds exactly correlates with the electronegativity of C, N, and O atoms (Pauling’s electronegativity C = 2.55, N = 3.04, O = 3.44) [10]. In Ba_2_SiO_4_ (BSO) structure, the SiO_4_ tetrahedrons are not only isolated, but also linked with Ba atoms via O atoms, providing two different Ba sites (I and II). The structural feature of BSO is very closely correlated with the evolution of the Si-2p core level in XPS spectra. 

The Si-2p XPS spectrum of BSO compound shows a broad and asymmetric band which can be deconvoluted into two peaks; at 100.4 eV and 102.4 eV [35,36]. It should be pointed out that the assignment of the binding energies (100.4 eV and 102.4 eV) can be easily understood by the chemical bond competition for Ba^2+^—[O–Si–O]^4−^—Ba^2+^. As presented in Figure 4, the Si‒O stretching IR mode of SiO_2_ appears around 1090 cm^-1^, and that of B_2_SiO_4_ formed by introducing Ba^2+^ ions into SiO_2_ matrix is around 900 cm^−1^. This IR spectral evolution between SiO_2_ and Ba_2_SiO_4_ reveals the weakness of the Si‒O bond by introducing Ba^2+^. Moreover, the Si‒O bond lengths of SiO_2_ and BSO are 1.620 Å and 1.665 Å (determined by Rietveld analysis), respectively. As indicated in Table 2, the average values of Ba(I)‒O and Ba(II)‒O bond length are 2.975 Å and 2.832 Å, respectively. As the Ba(II)‒O bond length is shorter than that of Ba(I)‒O, it might be assured that the chemical bond of Ba(II)‒O is somewhat stronger than that of Ba(I)‒O. Based on the bond competition for the bond character of Ba^2+^—[O–Si–O]^4−^—Ba^2+^, the Si‒O bond in the chemical bond of Ba(II)‒O‒Si is slightly weakened, which is associated with the lower shift of Si-2p binding energy. Thus, it is presumed that there are two distinct Si‒O sites (Ba(I)‒O‒Si and Ba(II)‒O‒Si), corresponding to 102.4 eV of Si-2p and 100.4 eV of Si-2p binding energy, respectively. For N^3−^-substituted BSON compound, the Si-2p spectrum shows a broad and asymmetric band which can be deconvoluted into three peaks; at 98.6 eV, 100.5 eV, and 102.4 eV. By comparison of the Si-2p binding energy between SiO_2_ and Si_3_N_4_, it is evident that the lowest binding energy (98.6 eV) of Si-2p is ascribed to the bond character of Si‒(O,N) formed by introduced N^3−^ ions into the SiO_2_ matrix, which can be proved by the N-1s binding energy at 399 eV in BSON compound.

### 3.5. Photoluminescence Spectra

**Figure 7:**
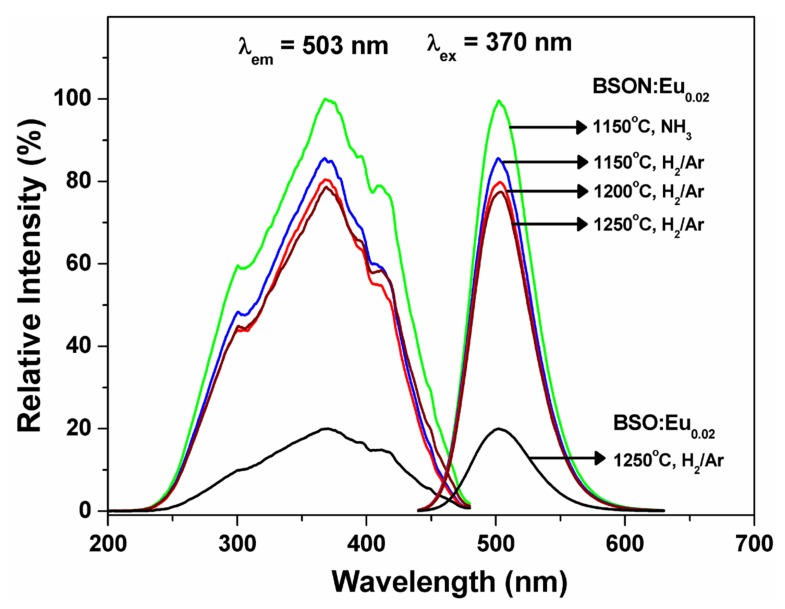
Photoluminescence (PL) spectra of BSO:Eu^2+^ and BSON:Eu^2+^ synthesized at different temperatures and atmospheres.

Figure 7 shows the PL spectra of BSO:Eu_0.02_ and BSON:Eu_0.02_ synthesized at different temperatures and atmospheric conditions.

**Figure 8:**
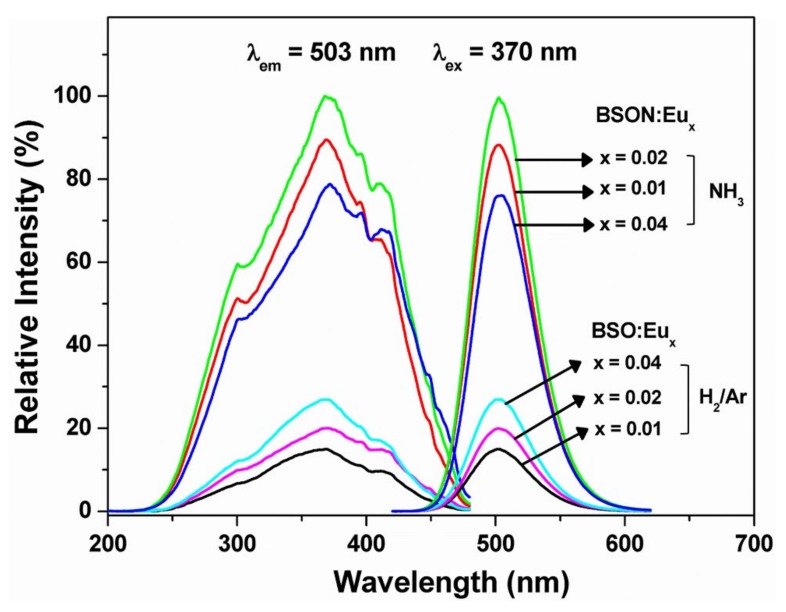
PL spectra of BSO:${\mathrm{Eu}}_{x}^{2+}$ (at 1250 °C under 4% H_2_–Ar) and BSON:${\mathrm{Eu}}_{x}^{2+}$ (at 1150 °C under NH_3_) phosphors synthesized with the variation of Eu content.

The highest PL intensity is shown from the BSON:Eu_0.02_ compound synthesized at 1150 °C under NH_3_ atmosphere. Figure 8 presents the PL spectra of BSO:Eu^2+^ and BSON:Eu^2+^ phosphors synthesized with different concentration of Eu.

**Figure 9:**
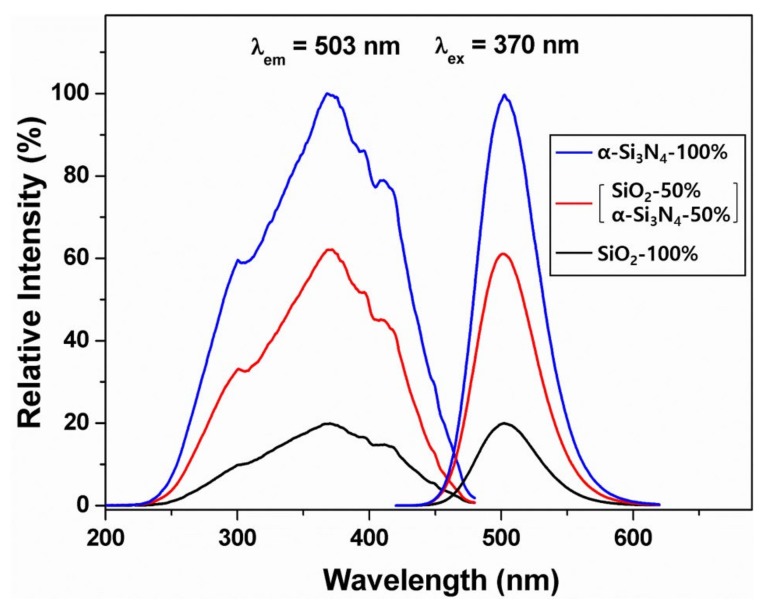
PL spectra of BSON:${\mathrm{Eu}}_{0.02}^{2+}\;$ phosphors prepared using α-Si_3_N_4_ and SiO_2_.

The excitation spectra of BSO:Eu and BSON:Eu phosphors measured at 503 nm are composed of bands between 200 nm and 475 nm, which could be attributed to the allowed 4f^7^(^8^S_7/2_)–4f^6^5d transitions of Eu^2+^ [37,38,39]. The emission spectra obtained under 370 nm excitation exhibit symmetric bands at 503 nm, corresponding to green-emission. Particularly, the full width at half maximum (FWHM) is about 50 nm for the BSON:Eu_0.02_ phosphor. Additionally, the emission intensity was increased with increasing Eu^2+^ content and the maximum intensity was observed at x = 0.02. The optimum Eu^2+^ content in BSON:Eux phosphors is about 0.02. In order to control the contents of N^3-^ ion in BSON:${\mathrm{Eu}}_{0.02}^{2+}$ phosphors, the weight ratio of starting materials, α-Si_3_N_4_ and SiO_2_, was changed. Figure 9 presents the PL spectra of BSON:${\mathrm{Eu}}_{0.02}^{2+}$ phosphor with different concentration of N^3−^ ions verified with SIMS: 0.064 (using only α-Si_3_N_4_), 0.035 (mixture of α-Si_3_N_4_ and SiO_2_, 1:1 Si atomic %), and 0.000 (using only SiO_2_).

**Figure 10:**
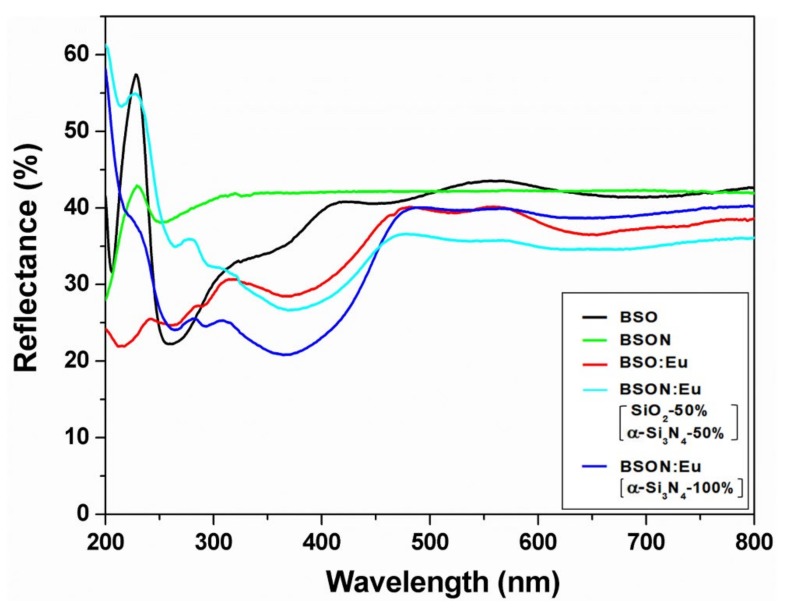
Diffuse reflectance spectra of BSO, BSO:Eu^2+^, BSON, and BSON:Eu^2+^.

There is no chemical shift in the PL spectra of three phosphors with different synthetic condition, the considerable change in the PL intensities, however, was observed. Surprisingly, the emission intensity of BSON:${\mathrm{Eu}}_{0.02}^{2+}$ phosphor synthesized (using only α-Si_3_N_4_) is about five times as high as that of BSO:${\mathrm{Eu}}_{0.02}^{2+}$ phosphor (using only SiO_2_). Additionally, the emission intensity of BSON:${\mathrm{Eu}}_{0.02}^{2+}$ phosphor synthesized (using mixture of α-Si_3_N_4_ and SiO_2_) is about three times as high as that of BSO:${\mathrm{Eu}}_{0.02}^{2+}$ phosphor (using only SiO_2_). The main question still remains about the difference of PL intensity in two phosphors, BSO:Eu^2+^ and BSON: Eu^2+^. According to all the above results together, it directly indicates that the remarkable enhancement of PL intensity could be ascribed to the substitute N^3−^ for O^2−^ ions in the Ba_2_SiO_4_ host lattice. Figure 10 shows the diffuse reflectance spectra (DRS) of BSO, BSON, BSO:Eu^2+^, and BSON:Eu^2+^.

When Eu^2+^ activators are introduced into the BSO host lattice, the bands appeared between 250 and 500 nm. It could be assured that the absorption bands between 300 and 500 nm of BSO:Eu^2+^ phosphor correspond to the 4f→5d transition of Eu^2+^, because the absorption band of BSO host lattice is around 270 nm. Particularly, the absorption intensity of BSON:Eu^2+^ (using only α-Si_3_N_4_) is highly increased with respect to that of the BSO:Eu^2+^ phosphor between 250 and 500 nm probably because of the N^3-^-substitution effect, which is in good agreement with PL results as shown in Figure 8. To verify the effect of N^3−^ substitution, the structural strain induced by N^3−^-doping was examined. The structural strain was calculated with Williamson−Hall (W−H) method [40]. At half-maximum intensity (*β_hkl_*), the total peak width was obtained from the sum of the size broadening (*β_D_*) and the strain broadening (*β_s_*):

*β_hkl_* = *β_D_* + *β_s_*, (1)

The size broadening is related to the Scherrer equation: *β_D_* = *kλ*/(*D* cos *θ*), where *D* is the crystallite size; *λ*, the wavelength of the X-rays; and k, the shape factor. The strain broadening is given by *ε* = *β_s_*/4tan *θ*, where ε is either compressive or tensile strain. Thus, the W−H equation from Equation (1) is obtained:

*β_hkl_* cos *θ/λ* = *k/D* + 4ε sin *θ/λ,* (2)

**Figure 11:**
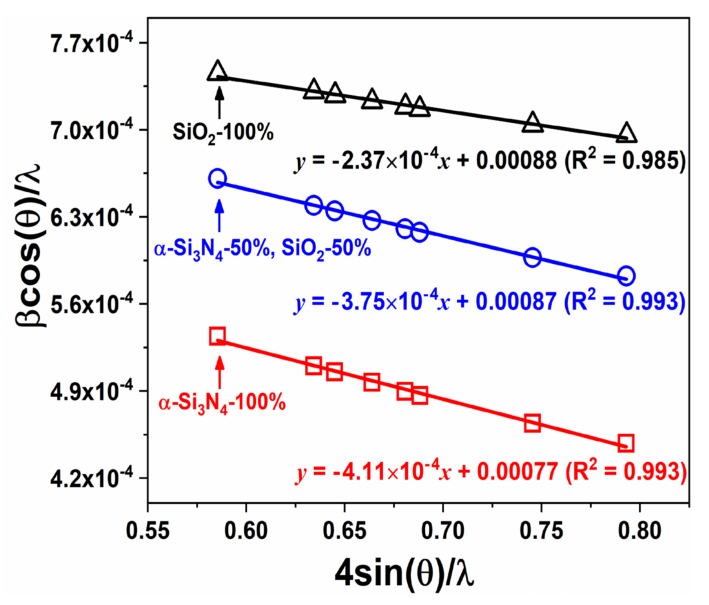
Williamson−Hall plots of BSON:${\mathrm{Eu}}_{0.02}^{2+}$ phosphors synthesized using α-Si_3_N_4_ and SiO_2_.

The plot of *β_hkl_* cos *θ/λ* vs. 4sin *θ/λ* allows us to estimate the structural strain by calculating slope of the graph and crystallite size from the y-intercept. The W−H plots present that the strain (*ε*) increases from −0.024% for BSO:Eu^2+^ (using only SiO_2_) to −0.038% for BSON:Eu^2+^ (using mixture of α-Si_3_N_4_ and SiO_2_) and it was further increased to −0.041% for BSON:Eu^2+^ (using only α-Si_3_N_4_) as shown in Figure 11.

**Figure 12:**
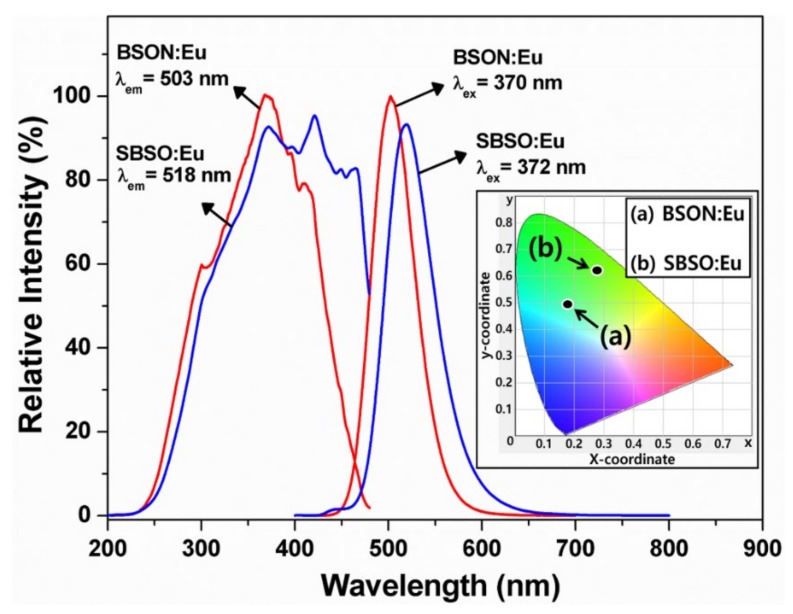
PL spectra of BSON:Eu^2+^ and a commercial (Sr,Ba)_2_SiO_4_:Eu^2+^ (SBSO:Eu) phosphor (obtained from Force4 Co., Ltd. in Korea). The inset shows CIE coordinates of BSON:Eu^2+^ and (Sr,Ba)_2_SiO_4_:Eu^2+^ phosphor.

The negative values indicate that there is compressive strain in the crystal lattice. Generally, with increasing reaction temperature, a crystal lattice expands and affects the tensile stress. Under the tensile stress, the crystal lattice undergoes non-radiative relaxation more easily in phosphors because of the lattice vibrations and/or the formation of defects [41,42,43]. Thus, the compressive stress occurred by the N^3−^ substitution compensates BSON:Eu^2+^ for thermally induced tensile stress and BSON:Eu^2+^ phosphor exhibits the enhanced PL intensity. Moreover, the structural strain effect by the N^3−^ substitution clearly appeared in DRS (see Figure 10). It is evident that for BSON:Eu^2+^, the intensified absorption bands in the range of 250~500 nm were shown after N^3−^ substitution. It is presumed that the compressive strain plays an important role in the PL intensity of the phosphors, while the difference of compressive strain before and after N^3−^ substitution is even very small. The luminescence behavior of BSON:Eu^2+^ with only α-Si_3_N_4_ was compared with a commercial (Sr,Ba)_2_SiO_4_:Eu^2+^ phosphor (obtained from Force4 Co., Ltd. in Korea) as indicated in Figure 12. 

**Figure 13:**
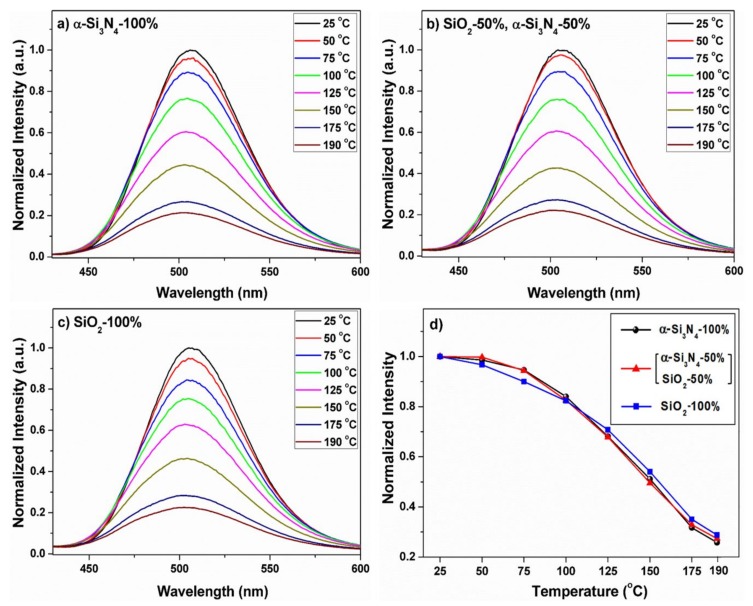
Temperature-dependent emission spectra of BSON:Eu^2+^ phosphor synthesized using α-Si_3_N_4_–100% (**a**), SiO_2_–50% and α-Si_3_N_4_–50% (**b**), SiO_2_–100% (**c**) and the variation of maximum emission intensity from room-temperature to 190 °C in the three phosphors (**d**). The PL intensity of phosphors at ambient temperature is fitted to 100%.

The PL spectra are quite similar. The emission spectra monitored under the characteristic excitations of BSON:Eu^2+^ and (Sr,Ba)_2_SiO_4_:Eu^2+^ phosphor exhibit a green-emission with different maximum wavelength. The emission intensity of BSON:Eu^2+^ is slightly higher than that of a commercial (Sr,Ba)_2_SiO_4_:Eu^2+^ phosphor. The Commission International de I’Eclairage (CIE) coordinates of BSON:Eu^2+^ and a commercialized (Sr,Ba)_2_SiO_4_:Eu^2+^ phosphor are as follows: for BSON:Eu^2+^, x = 0.177, y = 0.493; for (Sr,Ba)_2_SiO_4_:Eu^2+^, x = 0.276, y = 0.621 (see the inset in Figure 12). From the CIE coordinates, it is evident that the BSON:Eu^2+^ phosphor emission is more bluish-green compared with a commercialized (Sr,Ba)_2_SiO_4_:Eu^2+^. The emission spectra of BSON:Eu^2+^ and BSON:Eu^2+^ phosphors were measured from ambient temperature to 200 °C as shown in Figure 13. 

**Figure 14:**
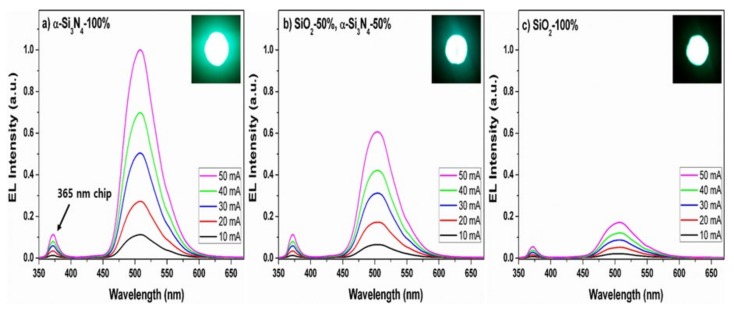
LED-PL spectra of NUV LEDs combined with of BSON:Eu^2+^ phosphor synthesized using α-Si_3_N_4_–100% (**a**), SiO_2_–50% and α-Si_3_N_4_-50% (**b**), SiO_2_–100% (**c**) monitored as a function of applied forward bias current.

The emission intensities at 150 °C greatly were decreased to about 40% of the initial one at ambient temperature, as previous research [44,45]. Kim et al. reported that thermal stability of Ba_2−x_Ca_x_SiO_4_:Eu^2+^ (x = 0.5) phosphor were highly increased in comparison to that of Ba_2_SiO_4_:Eu^2+^ phosphor [46]. Their results probably indicate that the thermal stability of phosphors is very closely related to the structural factor of the phosphors because Ba_2_SiO_4_:Eu^2+^ phosphor with orthorhombic phase (space group = *Pmcn*) [11] was transformed into the hexagonal phase (space group = *P*$\bar{3}$*m1*) [47] after partial Ca^2+^ substitution for Ba^2+^. As presented in Figure 13, the fact that there is no change in the thermal stability before and after N^3−^-substitution could strongly support that the primitive orthorhombic phase (space group = *Pmcn*) of Ba_2_SiO_4_:Eu^2+^ is preserved even after N^3−^ substitution in the Ba_2_SiO_4-δ_N_2/3δ_:Eu^2+^ phosphor. In order to determine whether or not promising candidate of the commercial use, three LEDs were prepared by combination of the three different phosphors and the 365 nm—emitting InGaN LED chip. Figure 14 shows the LED-PL spectra of BSON:Eu^2+^ synthesized (a) using only α-Si_3_N_4_, (b) BSON:Eu^2+^ synthesized using a mixture of α-Si_3_N_4_ and SiO_2_, and (c) BSO:Eu^2+^ synthesized using only SiO_2_ phosphor under forward bias currents between 10 and 50 mA. 

The LED-PL intensity of BSON:Eu^2+^ phosphor (see Figure 14a) is about five times as high as that of BSO: Eu^2+^ (see Figure 14c) at bias current 50 mA, which is well consistent with the PL result. It should be mentioned that the green-emitting BSON:Eu^2+^ phosphor could be considered as a breakthrough to overcome the disadvantages of the color rendering index for the conventional white UV-LEDs of YAG:Ce^3+^ phosphor base. Luminescent properties of BSON:${\mathrm{Eu}}_{0.02}^{2+}$ and BSO:${\mathrm{Eu}}_{0.02}^{2+}$ phosphors are compared in Table 3.

The *I_PL_* and *I_LED-PL_* ratio related to the radiative process have similar values, while the QY values related to the radiative and non-radiative process are a little different because the energy losses come from the non-radiative process. Regardless of the discrepancy between two-radiative (*I_PL_* and *I_LED-PL_*) and QY values, the luminescent properties of BSON:${\mathrm{Eu}}_{0.02}^{2+}$ phosphor reveal that the N^3−^-substitution allows us to develop high luminous and improved color rendering index phosphors for white NUV-LEDs.

## 4. Conclusions

In this paper, Ba_2_SiO_4-δ_N_2/3δ_:Eu^2+^ (BSON:Eu^2+^) phosphors with different N^3−^ contents were synthesized and characterized, resulting in superior luminous property and improved color rendering index for white NUV-LEDs. In the Eu^2+^-doped alkaline earth silicon-oxynitride phosphors, N^3-^ ion has a lower electronegativity and larger nephelauxetic effect than O^2−^ ion, providing more covalent bonding character between Eu and N atoms. Rietveld refinements reveal that N^3−^ ions are partially substituted for the O^2−^ ions in the SiO_4_-tetrahedra because the bond lengths of Si‒(O,N) (average value = 1.689 Å) are slightly elongated compared with those of Si‒O (average value = 1.659 Å), which results in the minute compression of the Ba(2)‒O bond lengths from 2.832 to 2.810 Å. IR and XPS measurement corroborate the Rietveld refinements; the appearance of the new IR mode at 850 cm^−1^ (Si‒N stretching vibration) and 98.6 eV (Si-2p binding energy) because of the introduced N^3−^ ions. Furthermore, in UV-region, the absorbance of N^3−^‒substituted BSON:Eu^2+^ (synthesized using only α-Si_3_N_4_) phosphor is approximately two times higher than that of BSO:Eu^2+^ (using only SiO_2_). The average N^3−^ content of BSON:Eu^2+^ (synthesized using only α-Si_3_N_4_) is determined to be ~0.06 using SIMS apparatus. Owing to the N^3−^ substitution, compensation of thermal annealing effect to some extent due to compressive strain occurred, the PL intensity of BSON:Eu^2+^ (synthesized using only α-Si_3_N_4_) is about five times as high as that of BSO:Eu^2+^ (using only SiO_2_). These phosphors could be a bridgehead for developing new phosphors and application in white NUV-LEDs field.

## Figures and Tables

**Table 1:** Structural parameters for BSO:Eu and BSON:Eu. Numbers in parentheses denote the standard deviation in the last digit value.

Compound	BSO:Eu	BSON:Eu
**Space Group**	***Pmcn***	***Pmcn***
**Crystal System**	Orthorhombic	Orthorhombic
***Z***	4	4
***a*** **(** **Å** **)**	5.809(6)	5.806(7)
***b*** **(** **Å** **)**	10.204(1)	10.198(2)
***c*** **(** **Å** **)**	7.500(6)	7.497(1))
***V*** **(** **Å** **^3^** **)**	444.65(1)	443.96(2)
**R_Bragg_ (%)**	5.33	9.37
**R_wp_ (%)**	10.6	13.9
**R_exp_ (%)**	6.07	6.85
***χ*^2^ (R_wp_^2^/R_exp_^2^)**	3.07	4.13

**Table 2:** Bond lengths (Å) and angles (°) for BSO:Eu and BSON:Eu.

Compound	BSO:Eu	BSON:Eu
**Ba1–O1**	2.591	2.542
**Ba1–O2 (× 2)**	2.918	2.922
**Ba1–O3 (× 2)**	2.941	2.921
**Ba1–O3 (× 2)**	3.009	3.051
**Ba1–O2**	3.101	3.132
**Ba1–O3 (× 2)**	3.160	3.121
**Average**	2.975	2.970
		
**Ba2–O3 (× 2)**	2.658	2.621
**Ba2–O2**	2.681	2.662
**Ba2–O2**	2.781	2.752
**Ba2–O3 (× 2)**	2.810	2.761
**Ba2–O1**	2.831	2.881
**Ba2–O1 (× 2)**	3.129	3.115
**Av** **erage**	2.832	2.810
		
**Si–O3 (× 2)**	1.636	1.681
**Si–O1**	1.681	1.752
**Si–O2**	1.682	1.642
**Average**	1.659	1.689
		
**O1–Si–O2**	111.1	112.2
**O1–Si–O3 (× 2)**	112.1	109.1
**O2–Si–O3 (× 2)**	107.2	109.1
**O3–Si–O3 (× 2)**	106.8	109.1

**Table 3:** Luminescent properties of phosphor materials.

Compound	Maximum *I_PL_* Ratio (%) ^1^	Maximum *I_LED-PL_* Ratio (%) ^1^, 50 mA	Quantum Yield (%)
BSO:${\mathrm{Eu}}_{0.02}^{2+}$	19	21	21
BSON:${\mathrm{Eu}}_{0.02}^{2+}$	100	100	71

^1^ PL and LED-PL intensity of BSON:${\mathrm{Eu}}_{0.02}^{2+}$ is fitted to 100% relative to that of BSO:${\mathrm{Eu}}_{0.02}^{2+}$.
